# Maternal reproductive history: trends and inequalities in four population-based birth cohorts in Pelotas, Brazil, 1982–2015

**DOI:** 10.1093/ije/dyy169

**Published:** 2019-03-18

**Authors:** Alicia Matijasevich, Cesar G Victora, Mariangela F Silveira, Fernando C Wehrmeister, Bernardo L Horta, Fernando C Barros, Pelotas Cohorts Study Group, Ana M B Menezes, Aluisio J D Barros, Andrea Dâmaso Bertoldi, Diego G Bassani, Helen Gonçalves, Iná S Santos, Joseph Murray, Luciana Tovo-Rodrigues, Maria Cecilia F Assunção, Marlos Rodrigues Domingues, Pedro R C Hallal

**Affiliations:** 1 Federal University of Pelotas, Brazil; 2 University of Toronto, Canada; 1 Department of Preventive Medicine, Faculty of Medicine FMUSP, University of São Paulo, São Paulo, Brazil; 2 Postgraduate Program in Epidemiology, Federal University of Pelotas, Pelotas, Brazil; 3 Postgraduate Program in Health and Behavior, Catholic University of Pelotas, Pelotas, Brazil

**Keywords:** Reproductive health, health equity, socioeconomic factors, cohort studies

## Abstract

**Background:**

Brazil experienced important progress in maternal and child health in recent decades. We aimed at describing secular trends as well as socioeconomic and ethnic inequalities in reproductive history indicators (birth spacing, previous adverse perinatal outcome, parity and multiple births) over a 33-year span.

**Methods:**

Four population-based birth cohort studies included all hospital births in 1982, 1993, 2004 and 2015 in Pelotas, Southern Brazil. Information on reproductive history was collected through interviews. Indicators were stratified by family income quintiles and skin colour. Absolute and relative measures of inequality were calculated.

**Results:**

From 1982 to 2015, the proportion of primiparae increased from 39.2% to 49.6%, and median birth interval increased by 23.2 months. Poor women were more likely to report short intervals and higher parity, although reductions were observed in all income and ethnic groups. History of previous low birthweight was inversely related to income and increased by 7.7% points (pp) over time—more rapidly in the richest (12.1 pp) than in the poorest quintile (0.4 pp). Multiple births increased from 1.7% to 2.7%, with the highest increase observed among the richest quintile and for white women (220% and 70% increase, respectively). Absolute and relative income and ethnic-related inequalities for short birth intervals increased, whereas inequalities for previous low birthweight decreased over time.

**Conclusions:**

In this 33-year period there were increases in birth intervals, multiple births and reports of previous low-birthweight infants. These trends may be explained by increased family planning coverage, assisted reproduction and a rise in preterm births, respectively. Our results show that socioeconomic and ethnic inequalities in health are dynamic and vary over time, within the same location.

**Key Messages**

- Along with an important decrease in fertility rates, median birth interval increased by 23 months and the proportion of primiparae increased from 39% in 1982 to nearly 50% in 2015.
- Although the frequency of short birth intervals (<36 months) was reduced over the period, women belonging to low-income groups still showed the highest values.
- Reports of previous preterm births increased over time and almost doubled among the poorest women, whereas reports of previous low-birthweight births increased mainly among the wealthiest.
- There was a steady increase in the incidence of multiple births in Pelotas, reaching higher levels than those reported for Brazil as a whole; the increase was restricted to high-income and to white women.
- Relative inequalities for short birth intervals, at least one previous pregnancy and multiple births increased, whereas those for low birthweight decreased over the study period.

## Introduction

Caring for and investing in womeńs and childreńs health are vital components of the right to health, encompassing reproductive, maternal, newborn and child health care.^1^ Maternal reproductive history, a known predictor of maternal health and pregnancy outcomes, should always be investigated during antenatal care, given its importance for guiding medical and therapeutic procedures. The most important indicators include birth spacing, previous adverse perinatal outcomes, parity and multiple births.

Birth spacing may be assessed through the time interval between delivery and conception of the next pregnancy (inter-pregnancy interval) or birth of the next child (birth interval), both of which substantially affect perinatal mortality, gestational age and birthweight, as well as the risk of pregnancy complications for mothers.^2–5^ Both short and long intervals, variously defined, have been associated with adverse outcomes, and a number of causal mechanisms were postulated,^2^ but the literature suggests that a single cut-off point of 36 months is adequate for predicting maternal and infant outcomes.^6^^,^^7^

The history of adverse perinatal events in a previous gestation increases the risk of developing an adverse outcome in subsequent pregnancies. History of a previous abortion, stillbirth, low birthweight and/or preterm birth are some of the best-documented indicators of adverse perinatal events. A recent systematic review showed almost a 4-fold increase of a new fetal death among women with a history of a previous stillbirth, compared with women with no such history.^8^ Subsequent pregnancies after a stillbirth or abortion—either spontaneous or induced—have been associated with increased risks of preterm and low birthweight births.^9^^,^^10^

The associations with parity vary according to the obstetric and neonatal outcomes under study. Compared with women with one to three previous births, the incidence of postpartum haemorrhage, preeclampsia, placenta praevia, macrosomia, post-date pregnancy and low Apgar scores is higher for grand multiparae (i.e. women having had four or more previous births).^11^ In contrast, neonatal and perinatal mortality are higher among babies born to nulliparous (i.e. women having no previous births) as well as to grand multiparous women, compared with those born to women with one to three previous deliveries.^12^

Multiple births are increasingly frequent, especially in high-income countries.^13^ These are associated with a number of complications both for the mother and fetus. Mothers suffer substantial morbidity due to the increased incidence in medical complications, and fetuses carry substantially higher risks of premature delivery, perinatal mortality and long-term neurodevelopmental impairments.^14^

In Brazil, maternal and child health outcomes are affected by the profound socioeconomic and ethnic group inequalities that characterize our society.^15–17^ For women, such inequalities affect their own health status—before, during and after pregnancy—directly and by limiting their access to and use of health services,^18^ and may be propagated in a transgenerational cycle.^19^ Research in such inequalities is needed, not only due to their effect in individual populations, but also because they are costly and burdensome to the entire health care system of a country.^20^

Brazil is the largest country in South America and the fifth largest country in the world.^21^ Its demographic transition began in the mid-1950s, since when there was an accelerated decline in population growth rates as a consequence of a steady reduction in fertility, from 6.1 children per woman in 1960 to 1.7 in 2015.^22^ The transition occurred concomitantly with cultural transformations, an intense process of industrialization, an increase of per capita income and education levels and a steady decline in infant mortality. Since the 1980s, Brazil also underwent positive changes in the social determinants of health and a universal health care system was created, with major improvements in maternal and child health indicators and in access to health care. Yet, important health inequalities still persist in the country.^17^

The city of Pelotas in southern Brazil is located in a relatively developed area of the country. It is a medium-sized city with around 340 000 inhabitants and a highly inequitable income distribution.^23^ During the years of 1982, 1993, 2004 and 2015, cohort studies including all births in the city were started, providing the opportunity to investigate how maternal reproductive history indicators (i.e. birth spacing, previous adverse perinatal outcome, parity and multiple births) evolved over time. The present study was aimed at describing secular trends for all women giving birth in the city, as well as for specific socioeconomic and ethnic groups, over a 33-year span.

## Methods

All women delivering in one of the Pelotas hospitals during the years of 1982, 1993, 2004 and 2015, and who were resident in the urban area of the city, were invited to participate in a population-based birth cohort study. Whereas in the 1982 and 1993 cohorts, infants who were less than 28 weeks of gestational age or weighed below 1000 g were excluded, in the two most recent cohort studies only newborns younger than 20 weeks or weighing below 500 g were excluded. Similar methodology was employed in all studies,^24–27^ including consistent variable definitions and comparable questions. The citýs maternity wards were visited on a daily basis, and mothers were interviewed at the hospital soon after delivery. Home births represented less than 1% of all births. Variables included in the present study were collected during the perinatal interview for the four cohorts.

Birth interval was defined as the number of months between the dates of the birth of the cohort child and the immediately preceding birth to the mother. Calculation was restricted to women with at least one previous live birth. Short birth intervals were defined as less than 36 months. Parity was defined as the number of times that a woman had given birth to a live-born baby (with any sign of life, irrespective of gestational age) or to a stillbirth (28 weeks of pregnancy or more), prior to the index pregnancy.

Women with at least one previous pregnancy were asked about abortions (induced or spontaneous), stillbirths and live births. If the pregnancy ended with a stillbirth or a live birth, it was enquired whether the outcome of the pregnancy was a preterm and/or low birthweight birth. Preterm birth was defined as a delivery before 37 completed gestational weeks, and low birthweight as less than 2500 g at birth. Information about previous preterm births was not available in the 1982 Pelotas cohort study.

In 1982 and 1993, maternal skin colour was classified according to the intervieweŕs observation, and in 2004 and 2015 was based on self-report. In 1982, only two categories were used, white or brown/black, whereas in the subsequent cohorts, three groups (as black, brown or white) were coded, according to the classification adopted by the Brazilian Census Bureau.^28^

Family income during the month preceding delivery of the index child was used as a measure of socioeconomic position. Family income was collected in Brazilian real and quintiles were calculated within each cohort. We refer to the first quintile (Q1) as the poorest quintile/poorest 20% and the fifth quintile (Q5) as the wealthiest quintile/wealthiest 20%.

Socioeconomic and ethnic inequalities in maternal reproductive history indicators (birth spacing, previous adverse perinatal outcome, parity and multiple births) were studied. In the present article we refer to inequalities as any measurable aspect of health that varies across individuals or groups, differentiating this term from inequities, which are systematic differences that are unfair and unjust.^29^

In order to investigate whether the effect of socioeconomic position on the outcomes varied over time, interactions between socioeconomic indicators (family income and maternal skin colour) and cohort year, fitted as an ordinal variable starting with 1982, for each of the reproductive outcomes were tested through logistic regression. Two indicators of economic-related inequality were estimated: (i) an indicator of absolute inequality, the slope index of inequality; and (ii) an indicator of relative inequality, the concentration index.^30^

The slope index of inequality is derived via regression of mean health outcome within a particular social group on the mean relative rank of social groups.^31^^,^^32^ In the case of quintiles of family income, each quintile included approximately 20% of the cohort, and midpoints of the quintile categories were calculated. The slope index of inequality was then obtained by regressing the outcomes studied on the midpoint score for each category. The slope of the regression line represents the absolute difference between the highest (score 1) and the lowest (score of 0) values of the socioeconomic position indicator.

The concentration index was calculated in its relative formulation, with no corrections.^33^ The concentration index uses an analogous approach to the Gini index, by ranking individuals according to socioeconomic position on the x-axis and for cumulative health condition on the y-axis. This index is expressed on a scale ranging from −100 to 100; a value of 0 represents perfect equality. If the outcome is more concentrated toward the richer groups, the concentration index assumes a positive value, as the curve is below the diagonal. When poorer groups are more affected than richer groups, the concentration index is negative.^33^^,^^34^

Ethnic group inequalities were studied using relative [i.e. ratio of (black plus brown) vs white] and absolute comparisons [i.e. the arithmetical difference between (black + brown) and white].

Data analyses included chi-square tests for heterogeneity and linear trends. As the intervals among the four cohorts are equal (11 years each), we used the *x*^2^ test for trend to compare the distribution of categorical outcome variables between cohort studies. This test was also used to analyse trends in reproductive outcomes within each category of family income and maternal skin colour over time. All analyses were performed with Stata V.14.0.^35^

Ethical approval for studies was not required in Brazil until 1996. The 2004 study was approved by the Ethics Committee of the School of Medicine and the 2015 by the School of Physical Education, Federal University of Pelotas, and written consent was obtained from the mothers.

## Results

Totals of 6011, 5304, 4287 and 4329 births were enrolled in the 1982, 1993, 2004 and 2015 birth cohort studies, respectively. The number of births decreased by 28% over the 33-year study period. Non-response rates at recruitment were below 2% in all cohorts.

Births occurring among primiparae, that is firstborn children, increased by 25% (from 39.2% in 1982 to 49.6% in 2015). As a consequence, the proportion of women with a previous reproductive history—having had at least one previous pregnancy—decreased by 17% (from 60.7% in 1982 to 50.4% in 2015) (Table 1).

**Table 1.** Time trends in reproductive history variables, Pelotas birth cohort studies

Variables	1982	1993	2004	2015	*P*-value
	*n* (%)	*n* (%)	*n* (%)	*n* (%)	
Parity					<0.001*
Primiparae	2357 (39.2)	1860 (35.1)	1684 (39.3)	2145 (49.6)	
1	1685 (28.0)	1471 (27.7)	1118 (26.1)	1330 (30.7)	
≥2	1967 (32.7)	1973 (37.2)	1484 (34.6)	852 (19.7)	
Median birth interval (months)^a^	35.0	48.3	55.0	58.2	<0.001*
Short birth interval (<36 months)^a^					<0.001
No	1736 (49.2)	1972 (63.8)	1647 (69.2)	1009 (70.6)	
Yes	1790 (50.8)	1118 (36.2)	733 (30.8)	421 (29.4)	
Previous preterm^a^					<0.001
No	NA	3060 (89.1)	1927 (80.2)	1756 (81.4)	
Yes		373 (10.9)	476 (19.8)	400 (18.6)	
Previous low birthweight^a^					<0.001
No	2767 (81.7)	2919 (85.3)	1932 (80.6)	1615 (75.2)	
Yes	620 (18.3)	503 (14.7)	465 (19.4)	533 (24.8)	
Multiple births					<0.001
No	5909 (98.3)	5223 (98.5)	4201 (98.0)	4213 (97.3)	
Yes	102 (1.7)	81 (1.5)	86 (2.0)	116 (2.7)	
Previous stillbirth^a^					0.410
No	3497 (95.8)	3349 (97.2)	2494 (95.8)	2108 (96.6)	
Yes	154 (4.2)	95 (2.8)	108 (4.2)	74 (3.4)	
Previous abortion^a^					0.067
No	2798 (76.7)	2474 (71.8)	1873 (72.0)	1752 (80.3)	
Yes	851 (23.3)	970 (28.2)	729 (28.0)	430 (19.7)	
Total of births	6011	5304	4287	4329	

NA, not available.

a Among women with at least one previous pregnancy.

*P*-value: *x*^2^ test for linear trend; *P-value: *x*^2^ test for heterogeneity.

The largest decline in reporting at least one previous pregnancy was observed among the richest women (32% reduction) and absolute and relative inequalities increased over time (Table 2). Black and brown women were more likely to report at least one previous pregnancy than white women in each cohort; however, inequalities remained stable over time (Table 3).

**Table 2.** Maternal reproductive history variables per cohort and family income quintile

Cohort study	Prevalence and CI 95%, per family income quintile					Slope index of inequality (95% CI)	Concentration Index (95% CI)
	Poorest	2nd	3rd	4th	Richest		
At least one previous pregnancy							
1982	63.8 (61.0; 66.5)	67.9 (65.2; 70.5)	57.1 (54.2; 60.0)	56.9 (54.0; 59.7)	58.0 (55.1; 60.8)	−11.1 (−15.4; -6.9)	−3.3 (−4.4; -2.1)
1993	70.0 (67.1; 72.8)	67.1 (64.4; 70.0)	63.9 (60.7; 67.0)	62.6 (59.6; 65.6)	60.2 (57.1; 63.2)	−11.2 (−15.6; -6.8)	−2.9 (−4.1; -1.8)
2004	62.6 (59.3; 65.8)	66.5 (63.3; 69.6)	61.7 (58.3; 65.1)	60.4 (57.0; 63.7)	52.0 (48.6; 55.5)	−13.1 (−18.2; -8.0)	−3.5 (−4.9; -2.1)
2015	56.4 (53.0; 59.7)	57.3 (54.0; 60.7)	52.8 (49.4; 56.2)	45.8 (42.5; 49.2)	39.7 (36.4; 43.0)	−21.7 (−26.7; -16.7)	−7.2 (−8.9; -5.5)
x^2^ test for linear trend	*P* <0.001	*P* <0.001	*P* = 0.097	*P* <0.001	*P* <0.001		
Median birth interval (months)^a^							
1982	14.0	34.0	36.0	40.0	37.0	8.8 (4.8; 12.8)	0.03 (0.02; 0.1)
1993	29.0	45.8	52.2	52.9	57.1	23.7 (18.5; 28.8)	0.06 (0.04; 0.07)
2004	43.0	47.9	55.9	63.7	69.0	31.8 (25.3; 38.4)	0.08 (0.06; 0.09)
2015	47.4	49.8	67.4	72.2	58.7	25.3 (15.9; 34.7)	0.06 (0.04; 0.08)
x^2^ test	*P* <0.001	*P* <0.001	*P* <0.001	*P* <0.001	*P* <0.001		
Short birth interval^a^							
1982	59.9 (56.2; 63.5)	52.0 (48.4; 55.5)	47.8 (44.0; 51.7)	44.6 (40.7; 48.5)	48.5 (44.7; 52.3)	−15.2 (−20.8; -9.5)	−4.8 (−6.7; -2.9)
1993	46.4 (42.6; 50.3)	39.1 (35.6; 42.8)	33.2 (29.2; 37.4)	32.0 (28.2; 36.1)	25.7 (22.0; 29.6)	−21.9 (−27.6; -16.1)	−10.0 (−12.7; -7.3)
2004	41.8 (37.5; 46.2)	34.8 (30.8; 39.0)	30.1 (26.0; 34.4)	23.3 (19.6; 27.4)	20.3 (16.4; 24.7)	−26.3 (−32.4; -20.1)	−13.8 (−17.2; -10.4)
2015	38.5 (33.2; 44.0)	33.0 (28.0; 38.4)	27.1 (22.1; 32.5)	19.3 (14.6; 24.9)	25.1 (19.7; 31.2)	−20.7 (−28.8; -12.6)	−11.4 (−16.0; -6.7)
x^2^ test for linear trend	*P* <0.001	*P* <0.001	*P* <0.001	*P* <0.001	*P* <0.001		
Previous preterm^a^							
1982	NA	NA	NA	NA	NA	–	–
1993	11.0 (8.8; 13.5)	11.9 (9.7; 14.3)	11.0 (8.6; 13.9)	10.6 (8.3; 13.2)	9.1 (6.9; 11.6)	−1.8 (−5.4; 1.7)	−2.5 (−7.9; 3.0)
2004	23.2 (19.6; 27.1)	20.3 (17.0; 23.9)	19.6 (16.1; 23.4)	20.4 (16.8; 24.3)	14.1 (10.7; 17.9)	−8.2 (−13.7; -2.7)	−5.4 (−10.0; -0.8)
2015	19.7 (16.2; 23.5)	21.9 (18.3; 25.8)	18.1 (14.6-22.0)	17.9 (14.3; 22.1)	13.5 (10.0; 17.6)	−7.3 (−12.9; -1.8)	−6.1 (−11.1; -1.1)
x^2^ test for linear trend	*P* <0.001	*P* <0.001	*P* = 0.001	*P* <0.001	*P* = 0.021		
Previous low birthweight^a^							
1982	28.6 (25.2; 32.1)	23.8 (20.8; 27.0)	14.9 (12.3; 17.9)	13.3 (10.7; 16.1)	10.0 (7.9; 12.6)	−23.5 (−28.0; -19.0)	−20.8 (−24.7; -16.8)
1993	18.1 (15.3; 21.1)	18.0 (15.4; 20.8)	14.5 (11.7; 17.7)	12.9 (10.4; 15.8)	8.1 (6.1; 10.5)	−11.2 (−15.3; -7.2)	−12.2 (−16.7; -7.7)
2004	24.8 (21.1; 28.7)	22.4 (19.0; 26.2)	18.1 (14.7; 21.9)	17.6 (14.3; 21.4)	11.7 (8.9; 15.3)	−14.6 (−20.0; -9.2)	−10.8 (−15.4; -6.2)
2015	29.0 (25.0; 33.3)	22.8 (19.1; 26.7)	23.5 (19.6; 27.7)	26.2 (21.9; 30.8)	22.1 (17.8-26.9)	−5.2 (−11.6; 1.2)	−4.4 (−8.6; -0.1)
x^2^ test for linear trend	*P* = 0.480	*P* = 0.984	*P* <0.001	*P* <0.001	*P* <0.001		
Multiple births							
1982	2.1 (1.4; 3.1)	1.0 (0.5; 1.7)	1.2 (0.6; 2.0)	2.1 (1.4; 3.1)	2.1 (1.4; 3.1)	0.5 (−0.7; 1.7)	5.7 (−6.7; 18.2)
1993	1.2 (0.7; 2.1)	1.2 (0.7; 2.0)	1.8 (1.0; 2.9)	1.6 (0.9; 2.5)	1.9 (1.1; 2.9)	0.8 (−0.4; 1.9)	8.8 (−3.6; 21.3)
2004	2.9 (1.9; 4.3)	1.6 (0.9; 2.7)	1.9 (1.1; 3.1)	1.4 (0.7; 2.4)	2.2 (1.3; 3.4)	−0.9 (−2.5; 0.7)	0.1 (−12.2; 12.5)
2015	1.8 (1.1; 3.0)	3.0 (2.0; 4.4)	2.1 (1.2; 3.3)	1.8 (1.1; 3.0)	4.6 (3.3; 6.3)	2.2 (0.4; 4.0)	10.2 (−0.8; 21.1)
x^2^ test for linear trend	*P* = 0.712	*P* <0.001	*P* = 0.105	*P* = 0.559	*P* = 0.001		

a Among women with at least one previous pregnancy.

*P*-value = *x*^2^ test for linear trend for reproductive outcomes within each category of family income over time.

**Table 3.** Maternal reproductive history variables per cohort and maternal skin colour

Cohort study	Prevalence and CI 95%, per maternal skin colour			Absolute inequality (PP) (brown + black) -white	Relative inequality (brown + black)/white
	White	Brown	Black		
At least one previous pregnancy					
1982	59.6 (58.3; 61.0)	66.0 (63.0; 68.8)		6.4	1.1
1993	63.6 (62.1; 65.1)	66.7 (60.3; 72.6)	70.3 (67.3; 73.1)	6.0	1.1
2004	58.1 (56.4; 59.9)	68.6 (63.0; 73.8)	67.3 (64.1; 70.4)	9.5	1.2
2015	49.1 (47.4; 50.9)	52.9 (48.7; 57.1)	54.5 (50.6; 58.3)	4.7	1.1
x^2^ test for linear trend	*P* <0.001	*P* <0.001	*P* <0.001		
Median birth interval (months)^a^					
1982	35.0	30.0		−5.0	0.9
1993	51.4	41.8	37.5	−13.3	0.8
2004	56.7	53.8	48.9	−6.6	0.9
2015	63.1	48.8	47.4	−15.3	0.8
x^2^ test	*P* <0.001	*P* = 0.110	*P* = 0.001		
Short birth interval^a^					
1982	48.9 (47.0; 50.7)	58.8 (55.0; 62.5)		9.9	1.2
1993	33.1 (31.2; 35.0)	40.1 (32.0; 48.7)	47.0 (43.0; 51.0)	12.6	1.4
2004	28.2 (26.1; 30.5)	32.6 (26.0; 40.0)	37.8 (33.7; 42.0)	8.2	1.3
2015	25.9 (23.2; 28.7)	38.9 (32.1; 46.1)	37.2 (30.9; 43.8)	12.1	1.5
x^2^ test for linear trend	*P* <0.001	*P* = 0.936	*P* = 0.001		
Previous preterm^a^					
1982	NA	NA	NA		
1993	10.5 (9.3; 11.7)	13.3 (8.4; 19.6)	11.8 (9.5; 14.5)	1.6	1.2
2004	18.5 (16.6; 20.4)	24.2 (18.4; 30.9)	22.3 (18.9; 26.0)	4.3	1.2
2015	17.7 (15.8; 19.8)	22.1 (17.6; 27.3)	19.1 (15.1; 23.6)	2.8	1.2
x^2^ test for linear trend	*P* <0.001	*P* = 0.053	*P* <0.001		
Previous low birthweight^a^					
1982	17.2 (15.8; 18.6)	23.2 (20.0; 26.7)		6.0	1.3
1993	13.9 (12.6; 15.3)	17.1 (11.6; 23.9)	17.1 (14.3; 20.1)	3.2	1.2
2004	18.0 (16.2; 20.0)	21.1 (15.6; 27.6)	23.0 (19.5; 26.7)	4.5	1.3
2015	24.2 (22.0; 26.4)	16.2 (12.2; 20.9)	34.8 (29.8; 40.0)	2.0	1.1
x^2^ test for linear trend	*P* <0.001	*P* = 0.627	*P* <0.001		
Multiple births					
1982	1.7 (1.3; 2.1)	1.9 (1.1; 2.8)		0.2	1.1
1993	1.5 (1.2; 1.9)	0.4 (0.01; 2.3)	1.9 (1.1; 2.9)	0.1	1.1
2004	1.9 (1.5; 2.5)	2.0 (0.7; 4.3)	2.3 (1.4; 3.5)	0.3	1.2
2015	2.8 (2.3; 3.5)	2.5 (1.4; 4.1)	2.1 (1.2; 3.5)	−0.5	0.8
x^2^ test for linear trend	*P* <0.001	*P* = 0.065	*P* = 0.652		

a Among women with at least one previous pregnancy.

*P*-value = *x*^2^ test for linear trend for reproductive outcomes within each category of maternal skin colour over time.

Even though the overall prevalence of short birth intervals decreased by half over time (Table 1), the poorest women and those with black or brown skin were more likely to report short birth intervals than the richest and white women in each cohort (Tables 2 and 3). Short birth interval prevalence declined faster among the latter groups, leading to an increase in absolute and relative inequalities over time.

History of a previous preterm birth increased by 70% between 1993 and 2015 (Table 1), being negatively associated with income in the 2004 and 2015 cohorts (Table 2). Whereas among poor women, reports of previous preterm births almost doubled, the increase among the wealthiest women was equal to 50%. The increase was also faster among black and brown women than among whites (Table 3). Income inequalities were small and increased discreetly over time, and ethnic inequalities remained almost stable (Tables 2 and 3).

History of a previous low birthweight birth showed a 35% increase in the whole population over the study period (Table 1), being inversely related to income in all cohorts (Table 2). Contrary to stable levels among brown women, there were increases of 40% among white and 200% among black women from 1993 to 2015 when information on the three skin colour groups was collected (Table 3). Also, levels were stable over time for the two poorest quintiles, but increased by 58%, 96% and 220% for those belonging to the 3th, 4th and 5th income quintile, respectively, in the period 1982–2015. Both absolute and relative inequalities decreased in the study period (Tables 2 and 3).

Multiple births showed a 60% increase between 1982 and 2015 (Table 1), mainly due to a 220% increase in the wealthiest women (Table 2). Absolute and relative economic inequalities also increased over time, with the highest inequalities observed in 2015, when multiple births were twice as common among the richest than among the poorest (Table 2). White women showed a 64% increase in multiple births, but no such trends were observed among black or brown women (Table 3). The magnitude of absolute and relative inequalities by ethnicity was low and remained stable over time.

History of previous stillbirth and abortion remained stable at approximately 4% and 25%, respectively (Table 1). Supplementary Tables 1 and 2, available as Supplementary data at *IJE* online, show that there were no time trends in income-related inequalities for these two outcomes. Reports of previous abortions were reduced over time for white women while remaining stable among black women, thus leading to increased absolute and relative ethnic inequalities (Supplementary Table 2, available as Supplementary data at *IJE* online).

Interactions between socioeconomic indicators (family income and maternal skin colour) and cohort year are described in Supplementary Table 3, available as Supplementary data at *IJE* online. Given the multiplicative nature of logistic regression, interaction tests refer to relative inequalities and are consistent with concentration index results. The presence of an interaction indicates that relative inequality changed over time, this being the case for birth intervals, at least one previous pregnancy and multiple births, where relative inequalities increased, and for previous low birthweight, where these decreased. The lack of evidence of an interaction between income and cohort year for previous stillbirths, previous preterm births and previous abortions is also in agreement with the lack of changes in relative inequalities over time. No interaction was observed between maternal skin colour and cohort year for most of maternal reproductive history outcomes, indicating that inequalities did not change over time.

## Discussion

During the study period, total yearly number of births dropped by 1800 and the median birth interval increased by 23.2 months. Low-income women were more likely to report short birth intervals and higher parity, but reductions over time were observed for all income and ethnic groups. Previous preterm births increased over time and almost doubled among the poorest women, whereas previous low birthweight births increased mainly among the wealthiest but to similar extent in all ethnic groups. The overall prevalence of multiple births increased, especially in the richest quintile and for white women. Absolute and relative income- and ethnic-related inequalities for short birth intervals increased, whereas inequalities for previous low birthweight decreased over time.

In the past three decades, Brazil experienced major political and economic changes that had a profound impact on the living conditions of its population. The country experienced economic growth up to 2011, and several social protection programmes targeting the poorest population groups were implemented. Coverage of essential maternal health interventions such as use of modern contraceptives, attendance at antenatal care and institutional delivery increased and equity improved dramatically, with the poorest 20% showing the fastest rates of improvement. Prevalence of modern contraceptive use increased from 57% in 1986 to 83% in 2013, and the family planning needs satisfied indicator—which excludes women who are willing to get pregnant—reached almost universal coverage.^36^

In spite of economic improvement in the country as a whole, the Pelotas region had slower growth than the rest of the country, as documented elsewhere.^23^ In 1982, the per capita gross domestic product of the city was similar to the national level, but dropped to 74% of the national value by 2015.^23^ Nevertheless, substantial improvements in maternal health and education were observed in the city during the course of the study period.^37^

Changes in reproductive histories over time reflected the sharp decline in fertility observed for Brazil as a whole.^17^ The crude birth rate for Pelotas fell from 23.1 to 12.9 births per 1000 population, and the annual number of births dropped in the same period in spite of population increase.^23^ Along with such a major decline in fertility, the proportion of primiparae increased from 39% to nearly 50% and the median birth interval increased by 0.7 months per calendar year. The decline in fertility is consistent with that observed for Brazil as a whole, where projected values indicate a total fertility rate of 1.69 children per woman for 2016.^38^ This process is directly related to the overall improvement of quality of life and educational attainment, reduction in child mortality, the rise in family planning with greater availability of contraceptive methods and the increasing participation of women in the labour market.^39^ The downturn in fertility rates did not occur uniformly among different socioeconomic groups of the population, being most marked for the wealthiest and for white women. Worldwide, fertility decline was universal, from an average of 4.5 births per woman in 1970–75 to 2.5 births per woman in 2010–15. The largest reductions took place in Asia.^40^

Birth intervals are also increasing globally. A study using data from demographic and health surveys (DHS) from 72 countries, ranging in date from 1985 to 2008, showed an overall median birth interval of 32.1 months, with marked heterogeneity between countries.^41^ Brazil, in 1996, had longer median birth intervals than the Latin American and Caribbean region, but still shorter than observed in the Pelotas 1993 cohort (31.5, 29.9 and 48.3 months, respectively). An analysis conducted in 66 countries between 1965 and 2014 showed that all major world regions had substantial increases in birth intervals. The largest increases were observed in sub-Saharan Africa and in Latin America and the Caribbean.^42^ Consistent with the international literature, birth intervals in our study increased markedly, mainly among the poorest; however, white and the wealthiest women still reported the longest birth intervals in 2015.^41^ Correspondingly, the frequency of short intervals (<36 months) declined sharply. Due to very well-known maternal and child health risks associated with short birth intervals,^3–5^^,^^43^ such a reduction can be regarded as a positive public health accomplishment.

The profile of Brazilian mothers has undergone significant changes over recent decades, with an increase in the percentage of mothers who start reproducing at later ages, a predominance of primiparous mothers and sustained increase in the rates of caesarean deliveries.^44^^,^^45^ Even though the proportion of adolescent mothers remained stable at around 15%, the percentage of mothers aged ≥30 years increased from about 25% in 1982 to almost 40% in 2015.^23^ Along with these changes, substantial improvements were observed in infant survival and—to a lesser extent—in maternal health indicators. In contrast to such improvements, there have been marked increases in preterm deliveries and stagnation in the prevalence of low birthweight.^17^ The city of Pelotas followed this trend, as described in the accompanying articles in this journal^23^^,^^44^ Specifically, preterm births rose from 6.3% in 1982 to 15.5% in 2015.^46^ The increased prevalence of histories of preterm births among the poor, and of low birthweight among the rich, are consistent with the results for the children born in the four cohorts^46^ and are likely related to changes in obstetric practices, particularly the remarkable increase in caesarean sections.

Rates of multiple births also vary considerably across the world. Among developed countries, twinning rates are between 2% and 4% of all births.^47^ Smits and Monden showed an average incidence of twinning across 75 low- and middle-income countries of 1.3%, with Benin and Vietnam being at the ends of the distribution (2.8% and 0.6%, respectively).^48^ Given the relative stability of monozygotic twinning rates across human populations, the variation observed among the countries is almost completely due to variation in dizygotic twinning.^49^ Recent decades have seen a major increase in the number and rate of multiple births in many developed countries.^13^^,^^48^ A combination of factors contributed to this increase, particularly the growing use of assisted reproductive technology, which is more likely to result directly in multifetal gestations, as well as in older age at the time of conception, when multifetal gestations are more likely to occur naturally.^50^ Among low- and middle-income countries, the changes in twinning rates over time have been small, suggesting that the influence of fertility treatments is still low in these countries.^50^ In Brazil, data from vital statistics showed a steady increase of multiple birth rates in recent decades, from 1.49% in 1994 to 2.09% in 2015.^51^ Our data from Pelotas not only confirm the national trends, albeit at higher rates than for Brazil as a whole, but also provide information on inequalities which are not readily available from secondary statistics. We showed that the increase in multiple births was restricted to high-income, white women, whereas levels remained stable for poor and for black women. This is certainly aligned to older age at the time of conception and the use of assisted reproductive technology.

The main strengths of our study include the use of consistently collected information from large population-based samples of women reflecting the socioeconomic spectrum in a middle-sized city, the high response rates and the availability of comparable variables in all four Pelotas cohort studies. Unfortunately, all outcome variables were assessed by maternal recall and as such may be affected by information bias. In addition, data on zygosity and use of fertility treatments were not available, preventing further exploration of the impact of these factors on multiple birth incidence in the city. The four cohorts are based on the date of delivery rather than the date of conception. This raises the possibility of fixed cohort bias,^52^ but given that none of the variables under study is affected by seasonality, it is unlikely that our results were affected.

In the 33 years covered by the Pelotas cohort studies, substantial progress was observed in maternal and child health indicators.^46^^,^^53–55^ Positive trends included reduced parity and increased birth intervals. On the negative side, reports of previous preterm and low birthweight deliveries became more frequent. Socioeconomic and ethnic group inequalities were narrowed down for some and increased for other indicators, but remain important for most indicators, indicating the need for further pro-equity interventions. Our results show that socioeconomic inequalities in health are dynamic, varying over time and between generations within the same city.

## Funding

The four cohorts received funding from the following agencies: Wellcome Trust, International Development Research Centre, World Health Organization, Overseas Development Administration of the United Kingdom, European Union, Brazilian National Support Program for Centres of Excellence (PRONEX), Brazilian National Council for Scientific and Technological Development (CNPq), Science and Technology Department (DECIT) of the Brazilian Ministry of Health, Research Support Foundation of the State of Rio Grande do Sul (FAPERGS), Brazilian Pastorate of the Child and Brazilian Association for Collective Health (ABRASCO).

## Pelotas Cohorts Study Group

Ana M B Menezes,^1^ Aluisio J D Barros,^1^ Andrea Dâmaso Bertoldi,^1^ Diego G Bassani,^2^ Helen Gonçalves,^1^ Iná S Santos,^1^ Joseph Murray,^1^ Luciana Tovo-Rodrigues,^1^ Maria Cecilia F Assunção,^1^ Marlos Rodrigues Domingues^1^ and Pedro R C Hallal.^1^

^1^Federal University of Pelotas, Brazil and ^2^University of Toronto, Canada.

**Conflict of interest:** None declared.

## Supplementary Material

Supplementary Tables

## References

[1] United Nations Economic and Social Council. CESCR General Comment No. 14: The Right to the Highest Attainable Standard of Health (Art. 12). New York, NY: ECOSOC, 2000.

[2] Conde-Agudelo A, Rosas-Bermudez A, Castano F, Norton MH. Effects of birth spacing on maternal, perinatal, infant, and child health: a systematic review of causal mechanisms. Stud Fam Plann 2012;43:93–114. 23175949 10.1111/j.1728-4465.2012.00308.x

[3] Conde-Agudelo A, Rosas-Bermudez A, Kafury-Goeta AC. Birth spacing and risk of adverse perinatal outcomes: a meta-analysis. JAMA 2006;295:1809–23. 16622143 10.1001/jama.295.15.1809

[4] Rutstein SO. Further Evidence of the Effects of Preceding Birth Intervals on Neonatal, Infant, and Under-five-Years Mortality and Nutritional Status in Developing Countries: Evidence from the Demographic and Health Surveys. Calverton, MD: ICF Macro, MEASURES DHS, 2008. 10.1016/j.ijgo.2004.11.012 15820369

[5] Wendt A, Gibbs CM, Peters S, Hogue CJ. Impact of increasing inter-pregnancy interval on maternal and infant health. Paediatr Perinat Epidemiol 2012;26:239–58. 22742614 10.1111/j.1365-3016.2012.01285.x PMC4562277

[6] Cleland J, Conde-Agudelo A, Peterson H, Ross J, Tsui A. Contraception and health. Lancet 2012;380:149–56. 22784533 10.1016/S0140-6736(12)60609-6

[7] Fotso JC, Cleland J, Mberu B, Mutua M, Elungata P. Birth spacing and child mortality: an analysis of prospective data from the Nairobi urban health and demographic surveillance system. J Biosoc Sci 2013;45:779–98. 22958417 10.1017/S0021932012000570 PMC3785173

[8] Lamont K, Scott NW, Jones GT, Bhattacharya S. Risk of recurrent stillbirth: systematic review and meta-analysis. BMJ 2015;350:h3080. 26109551 10.1136/bmj.h3080

[9] Makhlouf MA, Clifton RG, Roberts JM et al Adverse pregnancy outcomes among women with prior spontaneous or induced abortions. Am J Perinatol 2014;31:765–72. 24347257 10.1055/s-0033-1358771 PMC4061262

[10] Monari F, Pedrielli G, Vergani P et al Adverse perinatal outcome in subsequent pregnancy after stillbirth by placental vascular disorders. PLoS One 2016;11:e0155761. 27228078 10.1371/journal.pone.0155761 PMC4881909

[11] Babinszki A, Kerenyi T, Torok O, Grazi V, Lapinski RH, Berkowitz RL. Perinatal outcome in grand and great-grand multiparity: effects of parity on obstetric risk factors. Am J Obstet Gynecol 1999;181:669–74. 10486482 10.1016/s0002-9378(99)70511-9

[12] Bai J, Wong FW, Bauman A, Mohsin M. Parity and pregnancy outcomes. Am J Obstet Gynecol 2002;186:274–78. 11854649 10.1067/mob.2002.119639

[13] Collins J. Global epidemiology of multiple birth. Reprod Biomed Online 2007;15:45–52. 10.1016/s1472-6483(10)62251-1 18598609

[14] Blickstein I, Keith LG. Multiple Pregnancy. Epidemiology, Gestation and Perinatal Outcome. 2nd edn. Boca Raton, FL: Taylor & Francis, 2005.

[15] de Azevedo Barros MB, Lima MG, Medina LP, Szwarcwald CL, Malta DC. Social inequalities in health behaviors among Brazilian adults: National Health Survey, 2013. Int J Equity Health 2016;15:148. 27852275 10.1186/s12939-016-0439-0 PMC5112654

[16] Schmidt MI, Duncan BB, Azevedo e Silva G et al Chronic non-communicable diseases in Brazil: burden and current challenges. Lancet 2011;377:1949–61. 21561658 10.1016/S0140-6736(11)60135-9

[17] Victora CG, Aquino EM, Leal MC, Monteiro CA, Barros FC, Szwarcwald CL. Maternal and child health in Brazil: progress and challenges. Lancet 2011;377:1863–76. 21561656 10.1016/S0140-6736(11)60138-4

[18] Boccolini CS, Boccolini PMM, Monteiro FR, Venancio SI, Giugliani ERJ. Breastfeeding indicators trends in Brazil for three decades. Rev Saude Publica 2017;51:108. 29166437 10.11606/S1518-8787.2017051000029 PMC5697916

[19] Owen CM, Goldstein EH, Clayton JA, Segars JH. Racial and ethnic health disparities in reproductive medicine: an evidence-based overview. Semin Reprod Med 2013;31:317–24. 23934691 10.1055/s-0033-1348889 PMC4152894

[20] Zhang H, Rodriguez MR. Racial disparities in the risk of developing obesity-related diseases: a cross-sectional study. Ethn Dis 2012;22:308–16. 22870574

[21] UNICEF (United Nations Children’s Fund). UNICEF Data: Monitoring the Situation of Children and Women. New York, NY: UNICEF, 2017.

[22] The World Bank. *Fertility Rate, Total (Births per Woman)* 2018 https://data.worldbank.org/indicator/SP.DYN.TFRT.IN? (16 March, 2018, date last accessed).

[23] Bertoldi AD, Horta BL, Gonçalves H et al Trends and inequalities in maternal and child health in a Brazilian city: methodology and sociodemographic description of four population-based birth cohort studies, 1982–2015. Int J Epidemiol 2019;48(Suppl 1):i4–15. 30883654 10.1093/ije/dyy170 PMC6422064

[24] Hallal PC, Bertoldi AD, Domingues MR et al Cohort Profile: The 2015 Pelotas (Brazil) birth cohort study. Int J Epidemiol 2018;47:1048–h. 29126133 10.1093/ije/dyx219 PMC6124621

[25] Santos IS, Barros AJ, Matijasevich A, Domingues MR, Barros FC, Victora CG. Cohort Profile: The 2004 Pelotas (Brazil) birth cohort study. Int J Epidemiol 2011;40:1461–68. 20702597 10.1093/ije/dyq130 PMC3235016

[26] Victora CG, Barros FC. Cohort Profile: The 1982 Pelotas (Brazil) birth cohort study. Int J Epidemiol 2006;35:237–42. 16373375 10.1093/ije/dyi290

[27] Victora CG, Hallal PC, Araujo CL, Menezes AM, Wells JC, Barros FC. Cohort Profile: The 1993 Pelotas (Brazil) birth cohort study. Int J Epidemiol 2008;37:704–09. 17846051 10.1093/ije/dym177

[28] IBGE (Instituto Brasileiro de Geografia e Estatítica). Atlas do Censo Demográfico de 2010 [2010 Demographic Census Atlas]. Rio de Janeiro, Brazil: IBGE, 2010.

[29] Kawachi I, Subramanian SV, Almeida-Filho N. A glossary for health inequalities. J Epidemiol Community Health 2002;56:647–52. 12177079 10.1136/jech.56.9.647 PMC1732240

[30] Harper S, Lynch J. Methods for Measuring Cancer Disparities: Using Data Relevant to Healthy People 2010 Cancer-Related Objectives. Bethesda, MD: National Cancer Institute, 2005.

[31] Shaw M, Galobardes B, Lawlor D, Lynch J, Wheeler B, Davey Smith G. The Handbook of Inequality and Socioeconomic Position: Concepts and Measures. Bristol, UK: Policy Press, 2007.

[32] Wagstaff A, Paci P, van Doorslaer E. On the measurement of inequalities in health. Soc Sci Med 1991;33:545–57. 1962226 10.1016/0277-9536(91)90212-u

[33] Barros AJ, Victora CG. Measuring coverage in MNCH: determining and interpreting inequalities in coverage of maternal, newborn, and child health interventions. PLoS Med 2013;10:e1001390. 23667332 10.1371/journal.pmed.1001390 PMC3646214

[34] Barros AJ, Ronsmans C, Axelson H et al Equity in maternal, newborn, and child health interventions in Countdown to 2015: a retrospective review of survey data from 54 countries. Lancet 2012;379:1225–33. 22464386 10.1016/S0140-6736(12)60113-5

[35] StataCorp. Stata Statistical Software: Release 14.0. College Station, TX: StataCorp LP, 2015.

[36] Franca GV, Restrepo-Mendez MC, Maia MF, Victora CG, Barros AJ. Coverage and equity in reproductive and maternal health interventions in Brazil: impressive progress following the implementation of the Unified Health System. Int J Equity Health 2016;15:149. 27852276 10.1186/s12939-016-0445-2 PMC5112713

[37] Barros FC, Victora CG, Barros AJ et al The challenge of reducing neonatal mortality in middle-income countries: findings from three Brazilian birth cohorts in 1982, 1993, and 2004. Lancet 2005;365:847–54. 15752528 10.1016/S0140-6736(05)71042-4

[38] IBGE (Instituto Brasileiro de Geografia e Estatítica). *Projeção da população do Brasil por sexo e idade, para o período 2000/2060 [Projection of Brazilian population by sex and age for the 2000/2060 period]* 2018 https://sidra.ibge.gov.br/tabela/3727#resultado (6 March 2018, date last accessed).

[39] Anderson JSN, Schneider S. Brazilian demographic transition and the strategic role of youth. Espace Popul Soc. 2015 http://eps.revues.org/ (March 2018, date last accessed).

[40] United Nations, Department of Economic and Social Affairs, Population Division. World Fertility Report 2015 (ST/ESA/SER.A/415). New York, NY: UN, 2017.

[41] Rutstein SO. DHS Comparative Reports No 28: Trends in Birth Spacing. Calverton, MD: ICF Macro, 2011.

[42] Casterline JB, Odden C. Trends in inter-birth intervals in developing countries, 1965–2014. Popul Dev Rev 2016;42:173–94.

[43] Conde-Agudelo A, Rosas-Bermudez A, Kafury-Goeta AC. Effects of birth spacing on maternal health: a systematic review. Am J Obstet Gynecol 2007;196:297–308. 17403398 10.1016/j.ajog.2006.05.055

[44] Barros AJD, Horta BL, Wehrmeister FC et al Antenatal care and cesarean sections: trends and inequalities in four population-based birth cohorts in Pelotas, Brazil, 1982–2015. Int J Epidemiol 2019;48(Suppl1):i37–45. 30883657 10.1093/ije/dyy211 PMC6422067

[45] Brasil, Ministério da Saúde, Datasus. Saúde Brasil 2014: uma análise da situação da saúde e das causas externas. Capitulo 1: Como Nascem Os Brasileiros [Health Brazil 2014: An analysis of the health situation and external causes. Chapter 1: How Brazilians are born]. Brasilia: MS, 2015.

[46] Silveira MF, Horta BL, Menezes AMB et al Low birthweight and preterm birth: trends and inequalities in four population-based birth cohorts in Pelotas, Brazil, 1982–2015. Int J Epidemiol 2019;48(Suppl1):i46–53. 29939270 10.1093/ije/dyy106 PMC6422062

[47] Ananth CV, Chauhan SP. Epidemiology of twinning in developed countries. Semin Perinatol 2012;36:156–61. 22713495 10.1053/j.semperi.2012.02.001

[48] Smits J, Monden C. Twinning across the developing world. PLoS One 2011;6:e25239. 21980404 10.1371/journal.pone.0025239 PMC3182188

[49] American College of Obstetricians and Gynecologists, Society for Maternal-Fetal Medicine. ACOG Practice Bulletin No. 144: Multifetal gestations: twin, triplet, and higher-order multifetal pregnancies. Obstet Gynecol 2014;123:1118–32. 24785876 10.1097/01.AOG.0000446856.51061.3e

[50] Hall JG. Twinning. Lancet 2003;362:735–43. 12957099 10.1016/S0140-6736(03)14237-7

[51] Brasil, Ministério da Saúde, Datasus. SINASC - Sistema de Informações sobre Nascidos Vivos: nascimentos por residencia materna, tipo de gravidez e ano de nascimento 1994–2015 [Information system on live births: births by residence, type of pregnancy and year of birth 1994–2015]. Brasilia: MS, 2016.

[52] Strand LB, Barnett AG, Tong S. Methodological challenges when estimating the effects of season and seasonal exposures on birth outcomes. BMC Med Res Methodol 2011;11:49. 21501523 10.1186/1471-2288-11-49 PMC3102035

[53] Menezes AMB, Barros FC, Horta BL et al Stillbirth, newborn and infant mortality: trends and inequalities in four population-based birth cohorts in Pelotas, Brazil, 1982–2015. Int J Epidemiol 2019;48(Suppl1):i54–62. 30883653 10.1093/ije/dyy129 PMC6422061

[54] Santos IS, Horta BL, Menezes AMB et al Breastfeeding exclusivity and duration: trends and inequalities in four population-based birth cohorts in Pelotas, Brazil, 1982–2015. Int J Epidemiol 2019;48(Suppl1):i72–9. 30883659 10.1093/ije/dyy159 PMC6422059

[55] Wehrmeister FC, Horta BL, Matijasevich A et al Hospital admissions in the first year of life: inequalities over three decades in a Southern Brazilian city. Int J Epidemiol 2019;48(Suppl1):i63–71. 30883660 10.1093/ije/dyy228 PMC6422058

