# Supplementary material for: Maternal reproductive history: trends and inequalities in four population-based birth cohorts in Pelotas, Brazil, 1982–2015
Source: Int J Epidemiol. 2019 Mar 18;48(Suppl 1):i16–25. doi: 10.1093/ije/dyy169 (PMC6422066; doi:10.1093/ije/dyy169)
Supplement: Supplementary Tables [file dyy169_supplementary_tables.docx]

Supplementary Table 1. Previous stillbirth and abortion per cohort and family income quintile

| Cohort study | Prevalence and CI 95%, per family income quintile | | | | | Slope index of inequality (95% CI) | Concentration Index (95% CI) |
| --- | --- | --- | --- | --- | --- | --- | --- |
|  | Poorest | 2^nd^ | 3^rd^ | 4^th^ | Richest |  |  |
| ***Previous stillbirth***^§^ |  |  |  |  |  |  |  |
| 1982 | 4.4 (3.1;6.1) | 4.6 (3.3;6.3) | 5.5 (3.9;7.4) | 3.8 (2.5;5.5) | 2.8 (1.7;4.3) | -1.8 (-4,0;0,3) | -6.6 (-14.8;1,7) |
| 1993 | 3.7 (2.4;5.3) | 2.5 (1.5;3.8) | 1.7 (8.4;3.2) | 3.3 (2.1;5.0) | 2.1 (1.1;3.6) | -0.8 (-2.9;1.2) | -4.8 (-16.8;7.2) |
| 2004 | 5.6 (3.8;7.8) | 5.2 (3.5;7.3) | 4.1 (2.6;6.2) | 2.7 (1.5;4.5) | 2.8 (1.4;4.8) | -4.0 (-6.8;-1.3) | -14.2 (-24.5;-3.8) |
| 2015 | 4.1 (2.5;6.3) | 3.2 (1.8;5.2) | 4.4 (2.7;6.7) | 3.0 (1.6;5.2) | 1.7 (6.4;3.8) | -2.1 (-4.6;0.4) | -10.8 (-23.3;1.7) |
| *x^2^ test for linear trend* | *p=0.732* | *p=0.677* | *p=0.581* | *p=0.340* | *p=0.472* |  |  |

| ***Previous abortion***^§^ |  |  |  |  |  |  |  |
| --- | --- | --- | --- | --- | --- | --- | --- |
| 1982 | 22.6 (19.7;25.7) | 25.1 (22.1;28.2) | 21.4 (18.4;24.7) | 25.1 (21.9;28.5) | 22.2 (19.2;25.5) | -0.4 (-5.1;4.3) | 0.9 (-2.4;4.3) |
| 1993 | 26.7 (23.5;30.1) | 28.1 (25.0;31.3) | 28.4 (24.7;32.3) | 27.6 (24.1;31.2) | 31.7 (28.1;35.5) | 2.9 (-2.3;80.7) | 1.5 (-1.6;4.6) |
| 2004 | 27.0 (23.4;30.9) | 26.5 (22.9;30.3) | 27.1 (23.3;31.2) | 31.1 (27.1;35.3) | 28.7 (24.5;33.2) | 4.1 (-1.9;10.0) | 2.9 (-0.7;6.4) |
| 2015 | 19.7 (16.2;23.5) | 19.0 (15.6;22.7) | 20.4 (16.8;24.3) | 21.2 (17.2;25.5) | 18.4 (14.4;22.9) | 0.1 (-5.6;5.9) | -0.4 (-5.3;4.5) |
| *x^2^ test for linear trend* | *p=0.505* | *p=0.030* | *p=0.917* | *p=0.701* | *p=0.536* |  |  |

*Notes*: CI = confidence interval; ^§^ among women with at least one previous pregnancy; *p*-value = *x*^2^ test for linear trend for reproductive outcomes within each category of family income over time

Supplementary Table 2. Previous stillbirth and abortion per cohort and maternal skin colour

| Cohort study | Prevalence and CI 95%, per maternal skin colour | | | Absolute inequality (PP)  (Brown + Black) - White | Relative inequality  (Brown – Black) / White |
| --- | --- | --- | --- | --- | --- |
|  | White | Brown | Black |  |  |

| ***Previous stillbirth***^§^ |  |  |  |  |  |
| --- | --- | --- | --- | --- | --- |
| 1982 | 4.3 (3.6; 5.1) | 3.8 (2.5; 5.5) | | -0.5 | 0.9 |
| 1993 | 2.8 (2.2; 3.5) | 1.3 (0.2; 4.5) | 3.1 (1.9; 4.7) | -0.1 | 1.0 |
| 2004 | 3.6 (2,8; 4.6) | 5.4 (2.7; 9.4) | 5.3 (3.6; 7.5) | 1.7 | 1.5 |
| 2015 | 3.2 (2.4; 4.2) | 2.7 (1.2; 5.2) | 4.7 (2.8; 7.5) | 0.6 | 1.2 |
| *x^2^ test for linear trend* | *p=0.101* | *p=0.684* | *p=0.120* |  |  |
| ***Previous abortion***^§^ |  |  |  |  |  |
| 1982 | 23.7 (22.2; 25.3) | 21.6 (18.7; 24.8) | | -2.1 | 0.9 |
| 1993 | 28.9 (27.1; 30.6) | 32.3 (25.1; 40.2) | 24.6 (21.4; 28.0) | -2.9 | 0.9 |
| 2004 | 28.9 (26.8; 31.0) | 23.9 (18.2; 30.3) | 26.8 (23.2; 30.5) | -2.9 | 0.9 |
| 2015 | 18.4 (16.5; 20.4) | 22.9 (18.3; 28.1) | 22.6 (18.4; 27.3) | 4.4 | 1.4 |
| *x^2^ test for linear trend* | *p=0.023* | *p=0.041* | *p=0.660* |  |  |

*Notes*: CI = confidence interval; PP = percentage points; *p*-value = *x*^2^ test for linear trend for reproductive outcomes within each category of maternal skin colour over time; ^§^ among women with at least one previous viable pregnancy

Absolute inequality = absolute values of the arithmetic difference between (Brown + Black) and White categories

Relative inequality = ratio of (Brown + Black) *vs*. White categories

Supplementary Table 3. *P*-values for the interactions between SEP indicators and cohort year for each of maternal reproductive history outcomes

| Outcomes | Family income* | Maternal skin colour** |
| --- | --- | --- |
| At least one previous pregnancy | **<0.001** | 0.207 |
| Birth interval | **<0.001** | 0.338 |
| Short birth interval (<36 months) | **0.039** | 0.265 |
| Previous preterm | 0.865 | 0.888 |
| Previous low birthweight | **<0.001** | 0.170 |
| Multiple births | **0.017** | 0.424 |
| Previous stillbirth | 0.907 | 0.121 |
| Previous abortion | 0.703 | **0.037** |

*Notes*: SEP = socioeconomic position; * Family income variable was categorized in quintiles; ** Maternal skin colour was categorized into White and Black/Other
